# Validity of the Gender Dysphoria diagnosis and incidence trends in Sweden: a nationwide register study

**DOI:** 10.1038/s41598-021-95421-9

**Published:** 2021-08-09

**Authors:** Malin Indremo, Richard White, Thomas Frisell, Sven Cnattingius, Alkistis Skalkidou, Johan Isaksson, Fotios C. Papadopoulos

**Affiliations:** 1 Department of Neuroscience, Psychiatry, Uppsala University, Uppsala, Sweden; 2 Norwegian Institute of Public Health, Oslo, Norway; 3 Clinical Epidemiology Division, Department of Medicine Solna, Karolinska Institute, Stockholm, Sweden; 4 Institute of Women’s and Children’s Health, Obstetrics and Gynecology, Uppsala University, Uppsala, Sweden

**Keywords:** Epidemiology, Paediatric research, Diagnosis

## Abstract

The aim of this study was to examine the validity of the Gender Dysphoria (GD) diagnoses in the Swedish National Patient Register (NPR), to discuss different register-based definitions of GD and to investigate incidence trends. We collected data on all individuals with registered GD diagnoses between 2001 and 2016 as well as data on the coverage in the NPR. We regarded gender confirming medical intervention (GCMI) as one proxy for a clinically valid diagnosis and calculated the positive predictive value (PPV) for receiving GCMI for increasing number of registered GD diagnoses. We assessed crude and coverage-adjusted time trends of GD during 2004–2015 with a Poisson regression, using assigned sex and age as interaction terms. The PPV for receiving GCMI was 68% for ≥ 1 and 79% for ≥ 4 GD-diagnoses. The incidence of GD was on average 35% higher with the definition of ≥ 1 compared to the definition of ≥ 4 diagnoses. The incidence of GD, defined as ≥ 4 diagnoses increased significantly during the study period and mostly in the age categories 10–17 and 18–30 years, even after adjusting for register coverage. We concluded that the validity of a single ICD code denoting clinical GD in the Swedish NPR can be questioned. For future research, we propose to carefully weight the advantages and disadvantages of different register-based definitions according to the individual study’s needs, the time periods involved and the age-groups under study.

## Introduction

During the last decades, an increasing number of individuals with Gender Dysphoria (GD) have sought gender confirming health care^1,2^. According to the fifth version of the Diagnostic and Statistical Manual of Mental Disorders (DSM-5)^3^ GD is a condition that involves distress due to incongruence between an individual’s birth-assigned sex and gender identity. In the latest version of the International Classification of Diseases (ICD-11)^4^, Gender Incongruence has replaced the previous diagnoses Transsexualism and Gender Identity Disorders, and has been moved from the chapters of mental and behavioural disorders to a new chapter on conditions related to sexual health^5^. The DSM-5 term “Gender Dysphoria (GD)” will be mainly used throughout the article, except when referring to literature published when earlier versions of the DSM/ICD classification systems were in use.

Comparing estimates of prevalence and incidence rates of GD is challenging, due to differences in terminology, definitions, and methodology between studies^6^. In a meta-analysis, including a broad range of prevalence studies on transsexualism from Europe, USA and Australia, Arcelus et al.^1^ found an overall prevalence of 0.46/10,000; 0.68 for birth assigned males (aM) and 0.26 for birth assigned females (aF). The time trend analysis revealed an increase in reported prevalence over the last 50 years. The studies included in the meta-analysis used different means of identifying cases, including referrals to gender identity clinics, sex reassignment surgery, or gender confirming hormonal treatments, as well as medical records from governmental organizations and patient registers. In one of the largest cohort studies, including all individuals in the Netherlands assessed for GD between 1972 and 2015, the national prevalence in 2015 was estimated to 3.64/10,000 in aM and 1.93/10,000 in aF^7^.

In Sweden, previous incidence rates of GD have been based on the applications for change of legal sex and permission to undergo surgical sex reassignment 8, and on the registered ICD diagnoses in the Swedish Patient Register 9. The incidence of transsexualism, defined by applications for legal sex change and surgical sex reassignment, increased from 0.016 to 0.042/10,000 per year for aF and from 0.023 to 0.073/10,000 per year for aM from 1960 to 2010^8^. Regarding diagnoses in the Swedish registers, the Swedish National Board of Health and Welfare reported that 0.1/10,000 were diagnosed with GD for the first time in 2005 compared with 0.8/10,000 in 2015^9^. The most salient increase was visible among young individuals, 18–29 years of age.

These estimates do not necessarily reflect the prevalence of individuals who experience GD in the general population. In a population-based study conducted in Sweden in 2014, feeling like someone of a different gender was reported by 2.3%, whereas at least a partial desire for gender confirming hormones or surgery was reported by 0.5% of the participants^10^. In a previous study in the Netherlands, 0.6% of aM and 0.2% of aF reported an “incongruent gender identity” combined with a dislike of their body and a wish to obtain gender confirming hormones and/or surgery^11^. These findings vastly exceed previous incidence and prevalence estimates of GD, as defined by referrals, diagnoses, legal sex changes and received gender confirming treatments. Hence, the definition and inclusion criteria of GD greatly influence reports of incidence and prevalence trends, and may be confusing when not clearly stated.

### Aims of the study

Given the increase of GD the last decades, it is of crucial importance to correctly estimate the incidence and prevalence of GD in order to set priorities in health care development and policymaking. Even though official Swedish estimates employ data from the Swedish National Patient Register (NPR), no gold standard for a register-based definition of GD exists. The usefulness of diagnoses in patient registers for research purposes is dependent on diagnostic validity. The objectives of this study were to explore the validity of the GD diagnosis in the Swedish NPR, to discuss different register-based definitions of GD and to investigate incidence trends for GD in Sweden during 2001–2015.

## Results

We obtained data on all individuals aged 10 years or more at their first registered GD diagnosis from the NPR for the period 2001–2016. In order to discuss the most appropriate GD definition for the incidence calculations, we performed a register-based validation of the GD diagnosis during 2006–2014, including data from the Prescribed Drugs Register (PDR), which started in July 2005. We examined the impact of the coverage in the NPR on our definitions, as there has been underreporting of visits in specialized psychiatric outpatient care the first years of the study period. Crude and coverage-adjusted incidence rates of GD were calculated for the period 2004–2015, thus allowing for 1-year observation period after 2015.

### Coverage in the NPR

The coverage in the outpatient register for the first 3 years (2001–2003) varied from 8 to 18%. By 2010 the coverage had reached 61.5% and by 2015 93%. Details of the coverage in the NPR are provided in Supplementary Table S1.

### Validation of the GD diagnosis in the NPR

**Figure 1:**
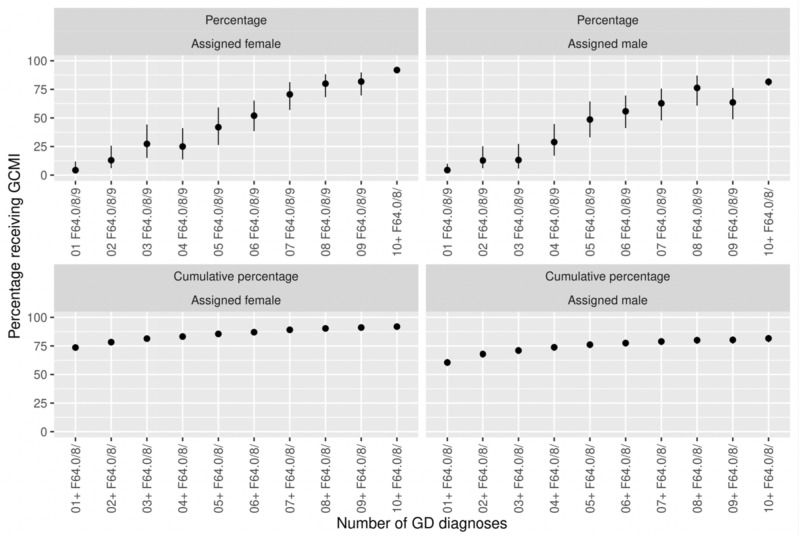
Gender confirming medical intervention (GCMI) by occurrence of F64 diagnoses in the NPR, 2006–2014. The upper graphs depict the occurrence of GCMI by exactly one, two etc. F64 diagnoses; the lower graphs depict the occurrence GCMI by at least one, two etc. F64 diagnoses.

Figure 1 displays the proportion of individuals (first diagnosis 2006–2014) that received any Gender Confirming Medical Intervention (GCMI), by number of occurrences of GD diagnoses before the end of follow-up in 2016. Of all individuals with ≥ 1 registered GD diagnosis, 33% did not acquire GCMI; 26% among aF and 40% among aM. In aF, 4% received GCMI by one single GD diagnosis, 25% by 4 GD diagnoses and 92% by ≥ 10 GD diagnoses. In aM the corresponding proportion was 4% by 1 single GD diagnosis, 29% by 4 GD diagnoses and 82% by ≥ 10 GD diagnoses. By using cumulative percentages, the proportion receiving GCMI by ≥ 4 GD diagnoses was 79% in total; 83% in aF and 74% in aM.

The validation analyses were then run in two separate time periods, to analyse whether the coverage would affect the proportion of individuals with GD diagnoses accessing GCMI. In the time period 2006–2009 the cumulative percentages receiving GCMI was slightly lower in all groups compared to the time period 2010–2014. By ≥ 4 diagnoses 76% of aF and 63% of aM received GCMI in 2006–2009; whereas 84% of aF and 76% of aM received GCMI by ≥ 4 diagnoses in 2009–2014. More details are displayed in Supplementary Table S2a–c.

From these results, we chose to use ≥ 4 GD diagnoses as an acceptable cut off for defining clinical GD, to further compare with alternative definitions. We chose to only display the incidence rates for the time period 2004–2015 as our main results, since the rates are too uncertain due to the low coverage in the NPR the first years of the study period. Crude and coverage-adjusted incidence rates for the whole period 2001–2015 are presented in Supplementary Materials.

### Incidence trends

**Figure 2:**
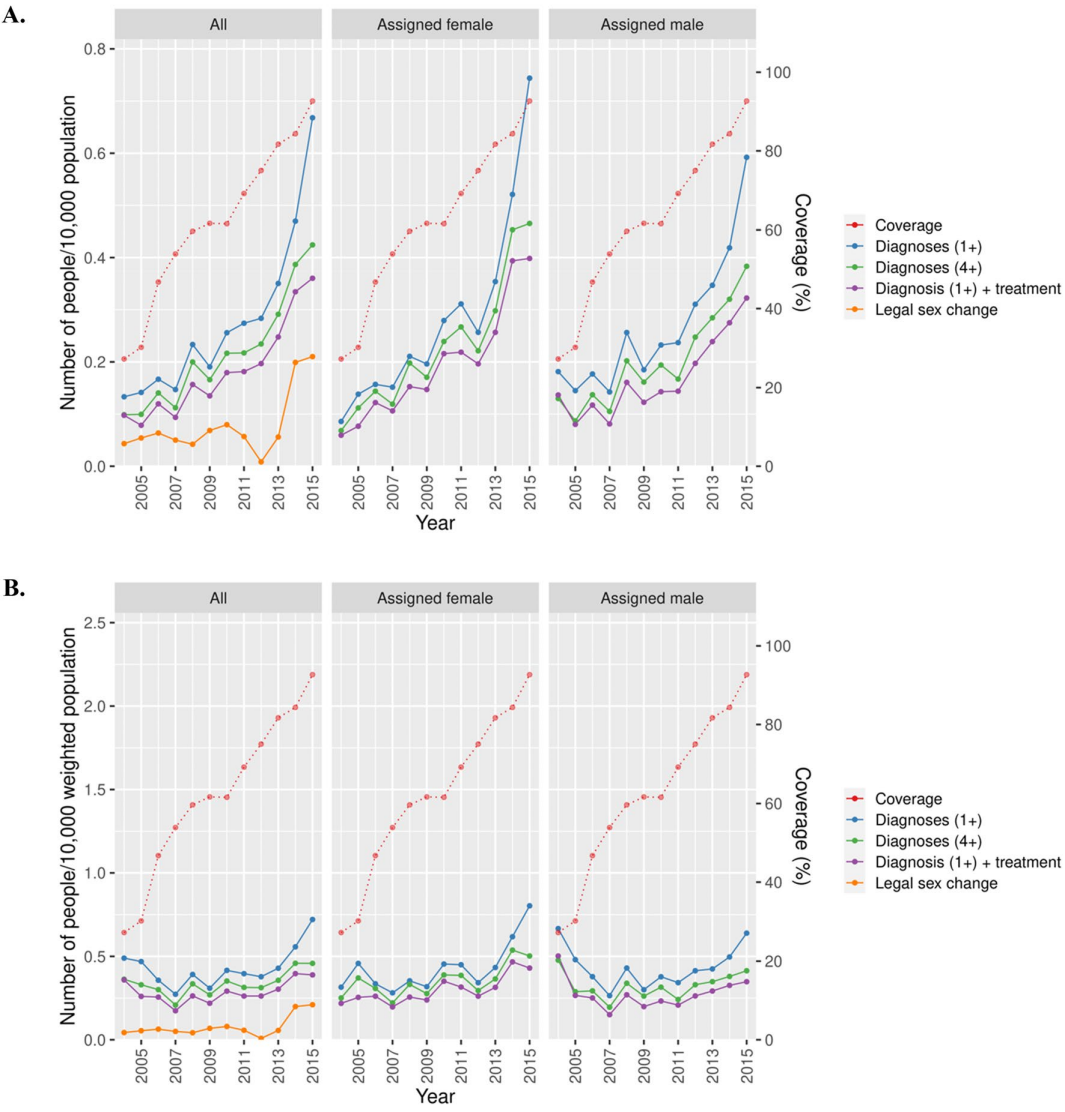
Crude (**A**) and coverage-adjusted (**B**) incidence rates of GD in Sweden 2004–2015 defined by ≥ 1 diagnosis, ≥ 4 diagnoses, ≥ 1 diagnosis with GCMI, stratified by birth assigned sex, along with legal sex rates and coverage in the NPR.

Figure 2A,B displays the crude and coverage-adjusted incidence of GD (first diagnosis 2004–2015) by different definitions; ≥ 1 GD diagnosis, ≥ 1 diagnosis with GCMI, ≥ 4 GD-diagnoses, and legal sex change. Independently of the definition, there was a notable increase of GD in both birth-assigned sexes during the observed time period. According to the ≥ 4 definition, the incidence of GD increased from 0.07 to 0.47 per 10,000 among aF and from 0.15 to 0.38 per 10,000 among aM. With the ≥ 1 GD definition, there was an increase from 0.09 to 0.74 per 10,000 among aF and from 0.18 to 0.59 per 10,000 among aM. Furthermore, the incidence of legal sex change followed a similar pattern, but was delayed with a few years from the first GD-diagnosis. There was a notable decrease of applications for legal sex change in 2010–2012.

**Figure 3:**
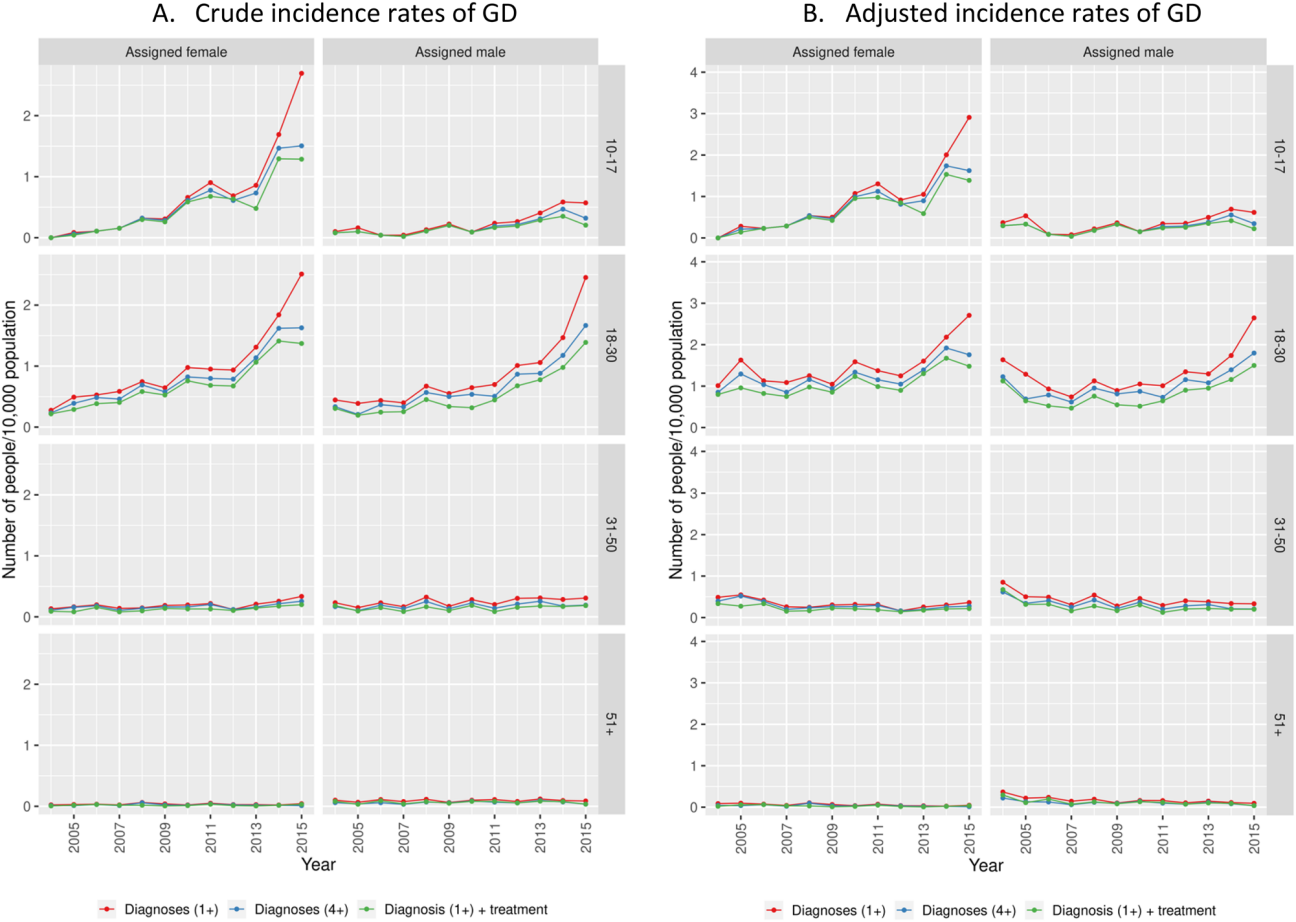
Crude (**A**) and coverage-adjusted (**B**) incidence rates of GD in Sweden 2004–2015, defined by ≥ 1 diagnosis, ≥ 4 diagnoses and ≥ 1 diagnosis with GCMI, stratified by birth-assigned sex and age.

Figure 3 displays the crude and coverage-adjusted assigned sex-specific incidence trends of GD further stratified by age. The differences between the definitions (≥ 1 GD diagnosis, ≥ 4 GD-diagnoses, and ≥ 1 diagnosis with GCMI) were visible in all age categories and time periods. The incidence of GD was on average 35% higher with the definition of ≥ 1 compared to the definition of ≥ 4 diagnoses. The discrepancies in the incidence rates depending on the different definitions used varied during the study period. In 2004 the overall incidence rate was 36% higher when using the ≥ 1 definition compared to ≥ 4 definition, and 57% higher in 2015. Details on differences between the definitions in crude incidence rates are available in Supplementary Table S2.

Kaplan Meier analysis showed that 50% of the individuals with ≥ 4 diagnosis had started GCMI within 1.3 years from first diagnosis; in three years the proportion increased to 75%, as displayed in Supplementary Fig. S1.

**Figure 4:**
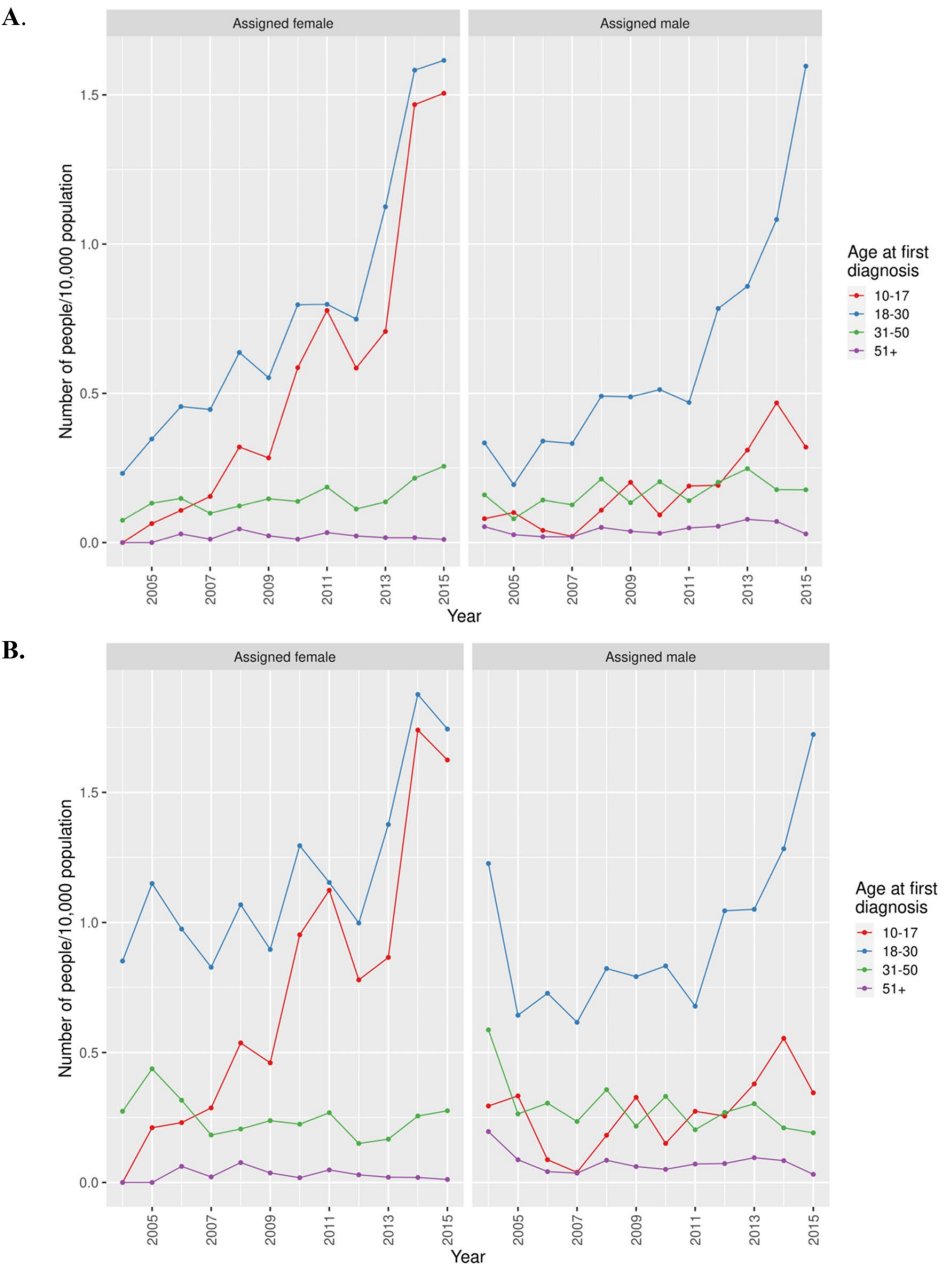
Crude (**A**) and coverage-adjusted (**B**) incidence rates of GD, defined by ≥ 4 diagnoses, in Sweden 2004–2015, stratified by birth assigned sex and age.

Figure 4 depicts the crude and coverage-adjusted incidence trends of GD, defined as ≥ 4 diagnoses, stratified by age and birth-assigned sex. In the groups where the incidence rates have increased the most, the crude incidence rates in 2015 for aF aged 10–17 and 18–30 years were 1.51 and 1.62 per 10,000 respectively. For aM, the corresponding incidence rates were 0.32 and 1.60 per 10,000. Table 1 displays the crude and coverage-adjusted incidence rate ratios (IRR) by sex and age groups as well as interaction terms for time trends and assigned sex, again defined as ≥ 4 GD diagnoses during 2004–2015. There were different time trends by age in aF and aM (interaction p-values < 0.01). We found different time trends by assigned sex only in the age categories 10–17 years. Among aF, the estimated linear effect sizes of time, IRR, were highest in the age categories 10–17 years and 18–30 years, with a 33% and 17% increase on average per year respectively. Similarly, the annual increase among aM was largest in the age groups 10–17 years and 18–30 years; 21% and 18% respectively. The increase was substantially lower in age categories 31–50 years and ≥ 51 years for both assigned sexes; for aF 7% and 3% and for aM 4% and 6% respectively. Adjusting for register coverage, the annual increase was reduced to 22% among aF 10–17 years old, and to 11% among aM; in the age category 18–30 years, the respective annual increases were after adjustment 8% among aF and 9% among aM. In the older age categories, there were no significant increases after adjusting for coverage.

**Table 1:** Crude and coverage-adjusted Poisson-derived incidence rate ratios (IRR) with 95% confidence intervals by sex and age groups as well as interaction terms for time trends and assigned sex, 2004–2015.

	Crude IRR	95% CI	p-value for interaction term time × assigned sex	Adjusted IRR	95% CI	p-value for interaction term time × assigned sex
**Age 10–17**			0.02			0.01
aF	1.33	1.27–1.39		1.22	1.17–1.28	
aM	1.21	1.13–1.29		1.11	1.04–1.18	
**Age 18–30**			0.62			0.61
aF	1.17	1.14–1.20		1.08	1.05–1.10	
aM	1.18	1.15–1.21		1.09	1.06–1.11	
**Age 31–50**			0.31			0.29
aF	1.07	1.03–1.12		0.98	0.94–1.02	
aM	1.04	1.01–1.08		0.95	0.91–0.99	
**Age 51+ **			0.65			0.64
aF	1.03	0.94–1.13		0.94	0.85–1.03	
aM	1.06	0.99–1.13		0.97	0.90–1.03	

Crude and coverage-adjusted incidence rates and incidence rate ratios (IRR) by sex and age groups as well as interaction terms for time trends and assigned sex, are presented for the whole time period 2001–2015 in Supplementary Fig. S2 and Supplementary Table S4.

## Discussion

In this registry-based study, we investigated the time trends of GD in Sweden during 2004–2015. For our purposes, we compared a definition of GD based on at least 4 diagnoses in the NPR, indicative of clinically relevant GD, with other registry definitions. The low coverage rate in the outpatient register in the NPR during the first years of the study, as well as changing clinical practices for GD over time add additional layers of complexity, and the estimated rates independent of definition should be interpreted with caution.

The validity of ICD-codes is a concern in all databases. As an attempt to validate the GD diagnoses included in the Swedish Registers, we used the occurrence of medical interventions as one proxy for a valid clinical GD diagnosis. We found that 79% of individuals with at least four GD diagnoses, also accessed GCMI. We abstained from including only individuals with GCMI for two reasons. Firstly, data on hormonal treatments from the PDR is only available from the mid 2005, thus projects assessing GD before that date cannot utilize this definition. Secondly, an individual with clinically significant GD may choose not to seek GCMI or the treatment may be withheld for various reasons. It may be hypothesized that individuals with 4 or more diagnoses who did not proceed with GMCI had indeed longstanding healthcare contact for gender incongruence-related symptoms. Possibly, excluding these individuals without GCMI would result in underestimating the incidence estimates of GD. When referring a patient to a gender clinic in Sweden, a GD diagnosis is most often registered by doctors outside the specialist teams, and may after assessment by a specialist team not be confirmed. Hence, including all individuals with a single GD diagnosis may result in overestimating the incidence trends, by including individuals without persisting GD. Our results align with previous research by Arnoldussen et al.^12^, who found that of the adolescents referred to the Amsterdam transgender clinic between 2001 and 2016, on average 85% were diagnosed with GD, out of which on average 77% continued with GCMI.

The choice to validate a diagnosis that has already been reconceptualized in the ICD-11 revision maybe questioned. Over the years, the medical diagnoses related to gender transition have been criticized for failing to encompass the whole spectrum of gender variance and to contribute to pathologizing and stigmatizing of non-normative gender identities and expressions^5,13–15^. An increasing visibility of different identifications, including non-binary or genderqueer gender identities, have been hypothesized to broaden the options for individuals with GD, including the option not to seek GCMI^16^. Our study did not aim to validate GD as a construct, but merely looking at ways to optimize the usefulness of GD registry diagnoses. However, we did find an increased proportion of people with GD accessing GCMI in the later period of our study, suggesting changes in the clinical practice, possibly with broader and more including criteria for GCMI.

Proposing a single best GD registry definition is complex. The number of registered diagnoses may be affected both by the coverage in the registers and the changing medical and coding practices in accordance to revised national and international guidelines. We provide data that equating a single GD diagnosis in the national registers to persistent GD can lead to misleading calculations on incidence rates. The degree of error can vary in different time periods and within age groups. For future research, we propose to carefully weight the advantages and disadvantages of different register-based definitions according to the individual study’s needs, the time periods involved and the age-groups under study.

### Incidence trends

To calculate incidence trends we chose to include individuals with four or more diagnoses in the registers as outlined above, in contrast to Swedish official statistics that include all individuals with a single GD diagnosis^9^. There was a clear trend of increasing incidence rates of GD in both aM and aF; GD increased eightfold in aF and almost doubled in aM between 2004 and 2015. Incidence rates of individuals changing their legal sex status also increased over time with a notable decrease of applications for legal sex change in 2010–2012. This coincides with a legal change, implemented in 2014, after which the previous prerequisite of being sterile in order to be able to apply for a new legal sex was removed. The trend of increasing incidence rates, especially among adolescents and young adults, was still present after adjusting for register coverage, though clearly flattened, especially among aM.

Whether the increased incidence rates could be explained by an actual increase in the prevalence of GD in the population or an increase in help seeking behaviour has been widely discussed^17,18^. Possible factors that might influence care seeking behaviour include increased availability of information and representation of transgender individuals and LGBTQ issues in the media, decreased stigmatization and increased social acceptance of different gender variations as well as more accessible health care. Suggested biological aetiologies that might affect GD have been investigated in areas such as structural neuroanatomy, genetics and exposure to prenatal androgens; however, the results as of yet are inconclusive and do not offer explanations to the increased incidence rates^17^.

It has also been hypothesized that the increase of GD would be explained by lower diagnostic thresholds^19^ and that new groups of individuals seek gender confirming health care, including individuals with more severe mental health issues and with less intense GD^20–22^. However, no major differences in demographic, psychological, diagnostic and treatment characteristics have been found in studies comparing patient cohorts over time, apart from a shift in sex ratio in favour of aF^12,23^. Mental health in individuals with GD seems to be multifactorial and influenced by experiences of stigma, social rejection, discrimination and cases of limited availability of transgender health care^19,24,25^. One could argue, that in a time of decreased stigmatization and more accessible health care for GD, an increase among young people would be expected, given that adolescence is a crucial time for identity formation^26^.

Regarding the shift in the sex ratio with a preponderance toward aF^12,22,23,27,28^, no definite explanations have been offered, even though several possible explanations have been discussed. In a British study, Aitken et al.^23^ conclude that the inversion of sex ratio in adolescents observed after 2006, appears to correspond with an increase in the number of clinic-referred youth with GD in general. Others hypothesize that parts of the increase of aF would be accounted for by a new group of aF with less intense GD^20^. Yet, no evidence has been presented to support that the increase in aF be accounted for by cases with lower degree of GD; on the contrary, patients referred the last years report the same high-level of GD as early referrals did^12,23^. Another hypothesis concerns that the earlier puberty onset in aF might have an impact on the increased numbers of adolescent aF coming forward, given that GD is often intensified during puberty^29^. However, as the increase of aF has been reported also among older adolescents, timing of puberty could only explain a smaller part of the increase^27^. More importantly, a number of studies have also suggested that there are greater social costs for aM to come out as transgender^23,27^, and that aM adolescents with GD are more often bullied because of their gender presentation, which may delay their process^22^.

It is imperative to point out that the increase of GD requires resources in health care development to meet the needs for this population. However, we need to assert caution on how to interpret the incidence estimates based only on one diagnosis in the official registers.

Our results point to the importance to acknowledge the variation behind the official statistics; that the population with a single GD in the Swedish registers includes individuals with varying degree of GD and needs of interventions. After the implementation of ICD-11 that is expected the coming years, coding practices will undoubtably change; hence a replicated validation study is essential.

### Strengths and limitations

Strengths of this study include access to a nationwide patient register^30^, in which we conducted a systematic and thorough investigation of trends in registered GD-diagnoses and coverage in the registers. We used a thorough methodology with proper exclusions and adjusting for register coverage, which we believe provides more accurate estimates of the incidence of GD derived from the national Swedish patient register.

There are a number of limitations in the study. The validity analysis would have been strengthened if supplemented with chart reviews, that could offer more information on the characteristics of individuals with registered GD diagnoses, including those not receiving any form of GCMI. The extremely low coverage in the NPR the first years of the study, was a major challenge for calculating accurate incidence rates for those years. The increasing register coverage over the remaining study period was taken into consideration by adjusting the rates for coverage and by doing sensitivity analyses on the validation part. In the latter, a different pattern was observed with a higher proportion of people diagnosed with GD receiving GCMI during the later period, which can be most probably interpreted as changes in the medical practices over time rather than a bias introduced by the less coverage in the early period of the study, as we would expect a higher proportion of treatment with the same number of registered diagnoses in the first period, which would correspond to more occurred but not registered visits.

## Method

### Swedish registers

Register data was collected from Statistics Sweden (SCB) and The Swedish National Board of Health and Welfare. All registers use the 10-digit National Registration Number (NRN), a unique personal identifier assigned to all Swedish residents, which allows linkage between registers. When retrieving data from the registers, the NRNs are, to secure anonymity, replaced with other unique numbers, which cannot be linked to other data sources. We retrieved data from the National Patient Register (NPR)^30^, comprising information on primary and secondary ICD diagnoses from visits to specialist outpatient health care since 2001 and from inpatient care since 1964 (nation-wide coverage since 1987), including surgeries, admission and discharge dates, as well as data from the PDR on redeemed medication since July 2005^31^.

While the coverage of the inpatient register is well documented, the coverage of the outpatient register is less well known. In order to quantify the coverage of the outpatient register during the study period, additional data on total numbers of conducted psychiatric health care visits were collected from the Swedish association of local authorities and regions (SKR) and were compared to the registered visits in the outpatient register, with or without a registered diagnosis, as not all registered visits have a registered diagnosis. The aggregated data on registered visits and diagnoses were provided by the National Board of Health and Welfare.

Sociodemographic data on birth dates and assigned sex at birth were retrieved from the Swedish Total Populations Register^32^. Aggregated yearly data on change of legal sex applications was provided by the National Board of Health and Welfare, with full national coverage during the whole study period. The study was approved by the Central Ethical Review Board in Stockholm (Dnr Ö30-2016). All methods were performed in accordance with the relevant guidelines and regulations.

### Definitions

#### Gender Dysphoria

ICD-10 codes used to classify GD during the study period were F64.0 (transsexualism), F64.8 (other gender identity disorders) and F64.9 (gender identity disorder, unspecified). The following definitions were compared and assessed: ≥ 1 registered GD diagnosis in the NPR; different numbers of registered GD diagnoses; ≥ 1 registered GD diagnosis and gender confirming medical intervention (GCMI); and legal sex change.

#### Gender confirming medical interventions

Gender confirming hormonal treatment was identified in the PDR using the ATC codes for testosterone (G03B) for aF; antiandrogens and estrogen (G03C, L02AA, G03D, L02AB, G03H, L02BB, G04CB, C03DA01, L02AE, H01CA) for aM. Puberty blockers (L02AE, H01CA) were identified independent of assigned sex for individuals up to 18 years. Surgical gender confirming treatments were identified in the NPR using the following ICD-codes: mastectomy and breast reductions (HAC10, HAC15, HAC20, HAC99, HAD20, HAD30, HAD35, HAD99, HAE99) and genital surgeries (KFH50, KGV30, KGW96, KGH96, LCD00, LCD01, LCD04, LCD10, LCD11, LCD96, LCD97, LED00) in aF; breast reconstruction (HAD00, HAD10, HAD99, HAE00, HAE20, HAE99), genital surgeries (LEE10, LEE40, LEE96, LFE10, LFE96 and KGC10) and larynx surgery (DQD40) in aM.

#### Study period

Data on GD diagnoses and surgical interventions were available between January 1st, 2001 and December 31st, 2016, while data on hormonal treatments were available from the PDR from July 1st, 2005 to December 31st, 2016. For our validation study we limited the time frame for first GD diagnosis in the NPR to the period from January 1st, 2006 to December 31st, 2014. The validation study period was initiated six months after the start of PDR and terminated two years before the end of the study period. Given that the waiting lists and the evaluation process leading up to hormonal treatment and/or surgery could last up to at least two years, including the two latter years would risk GCMI misclassification. We included GCMI registered between January 1st, 2006 and December 31st, 2016. Incidence rates as the main results were displayed for the years 2004–2015, due to the low coverage in the NPR the first years of the study period.

### Procedure

Between 2001 and 2016, 4378 individuals in Sweden received at least one GD diagnosis. We excluded individuals who had been diagnosed with the previous ICD-8 and ICD-9 GD diagnoses (n = 100), or had previously changed legal sex (n = 22), given that these were not incident cases. Using the NPR and PDR, individuals with surgical or prescribed hormonal gender confirming treatment prior to first diagnosis were similarly excluded (n = 166). From the remaining 4090 individuals, two overlapping datasets were extracted: one for the validation analyses (2006–2014, n = 2083) and one for the incidence trends (2004–2015, n = 3191).

### Statistical analyses

#### Validation of the GD diagnoses

There is no gold standard for a register-based definition of GD. We therefore evaluated the positive predictive value (PPV) of the register-based diagnoses by estimating the proportion of individuals who received GCMI within at least 2 years of follow-up time. Given that GCMI is included in the recommended protocol for GD, we used such treatment as a proxy for enduring GD, requiring medical interventions. We used increasingly strict requirements on the definition of GD: requiring one, two, three, four, etc. registered GD diagnoses. As a sensitivity analysis, we repeated the validation analyses in two separate time periods; 2006–2009 and 2010–2014, to examine if differences of coverage in the NPR had an impact on the results. The time between first diagnosis and commencement of treatment were calculated by using Kaplan Meier estimator.

#### Incidence trends

To visualize and compare the time trends, incidence rates of GD were calculated and plotted using the above-mentioned definitions of GD: ≥ 1 registered GD diagnosis in the NPR; x number of registered GD diagnoses; ≥ 1 registered GD diagnosis and GCMI; and legal sex change applications. To clarify the uncertainty in the estimates, the yearly coverage in the NPR was plotted in the incidence rates figures. Incidence rates adjusted for coverage were also calculated by dividing the rates with the yearly factor of coverage (ie. adjusted rate = rate/(0.20), for a coverage of 20%).

Using our proposed GD definition, incidence rates were estimated and displayed. To investigate the time trends and the differences in trends between age and sex categories, we used multiple Poisson regression models, with “diagnoses per year” as outcome. To identify if the trends within aF/aM were significantly different by age, we ran two models, with aF and aM data separately, using the following exposures: time + age and time + age + time × age. We compared the models using a likelihood ratio test (LRT). We then investigated if the trends within the age groups were significantly different according to assigned sex using the same logic. Within each age group, we ran the two models, using age-specific stratified data, with the following exposures: time + sex and time + sex + time × sex and compared the models. If the LRT was not significant, we ran a model in a dataset that was aggregated over assigned sex with time as exposure. If the LRT was significant, we ran two extra models: time within aF and time within aM and reported the coefficients and 95% confidence intervals from the relevant model. We also run the models adjusted for the coverage rate in the outpatient register. All tables and statistical analyses were generated in the software package R^33^. Data.table v1.12.2 was used for data cleaning, aggregations, and summary statistics and ggplot2 v3.2.0 was used for producing figures^34^.

## Supplementary Information

Supplementary Information.

## Data Availability

Our study includes data from Swedish health care registers, which cannot be shared due to confidentiality issues. Data are available from the National Board of Health and Welfare in Sweden and Statistics Sweden (https://www.scb.se/om-scb/kontakta-oss/statistikservice/fraga-oss/registerservice@socialstyrelsen.se).
