# Supplementary material for: Validity of the Gender Dysphoria diagnosis and incidence trends in Sweden: a nationwide register study
Source: Sci Rep. 2021 Aug 9;11:16168. doi: 10.1038/s41598-021-95421-9 (PMC8352918; doi:10.1038/s41598-021-95421-9)
Supplement: Supplementary file 1 — Supplementary Information. [file 41598_2021_95421_MOESM1_ESM.docx]

**Validity of the Gender Dysphoria diagnosis and incidence trends in Sweden: a nationwide register study**

Authors

Malin Indremo^1^*, Richard White^2^, Thomas Frisell^3^, Sven Cnattingius^3^, Alkistis Skalkidou^4^, Johan Isaksson^1^, Fotios C Papadopoulos^1^.

Affiliations:

^1^ Department of neuroscience, Psychiatry, Uppsala University, Uppsala, Sweden.

^2^ Norwegian Institute of Public Health, Oslo, Norway.

^3^ Clinical Epidemiology Division, Department of Medicine Solna, Karolinska Institute, Stockholm, Sweden.

^4^ Institute of Women's and Children's Health, Obstetrics and Gynecology, Uppsala University, Uppsala, Sweden.

Correspondence to: [malin.indremo@neuro.uu.se](mailto:malin.indremo@neuro.uu.se)

**Supplementary Figures and Tables**

**Supplementary Figure 1.** Kaplan-Meier curve for years from first GD diagnosis to commencement of GCMI


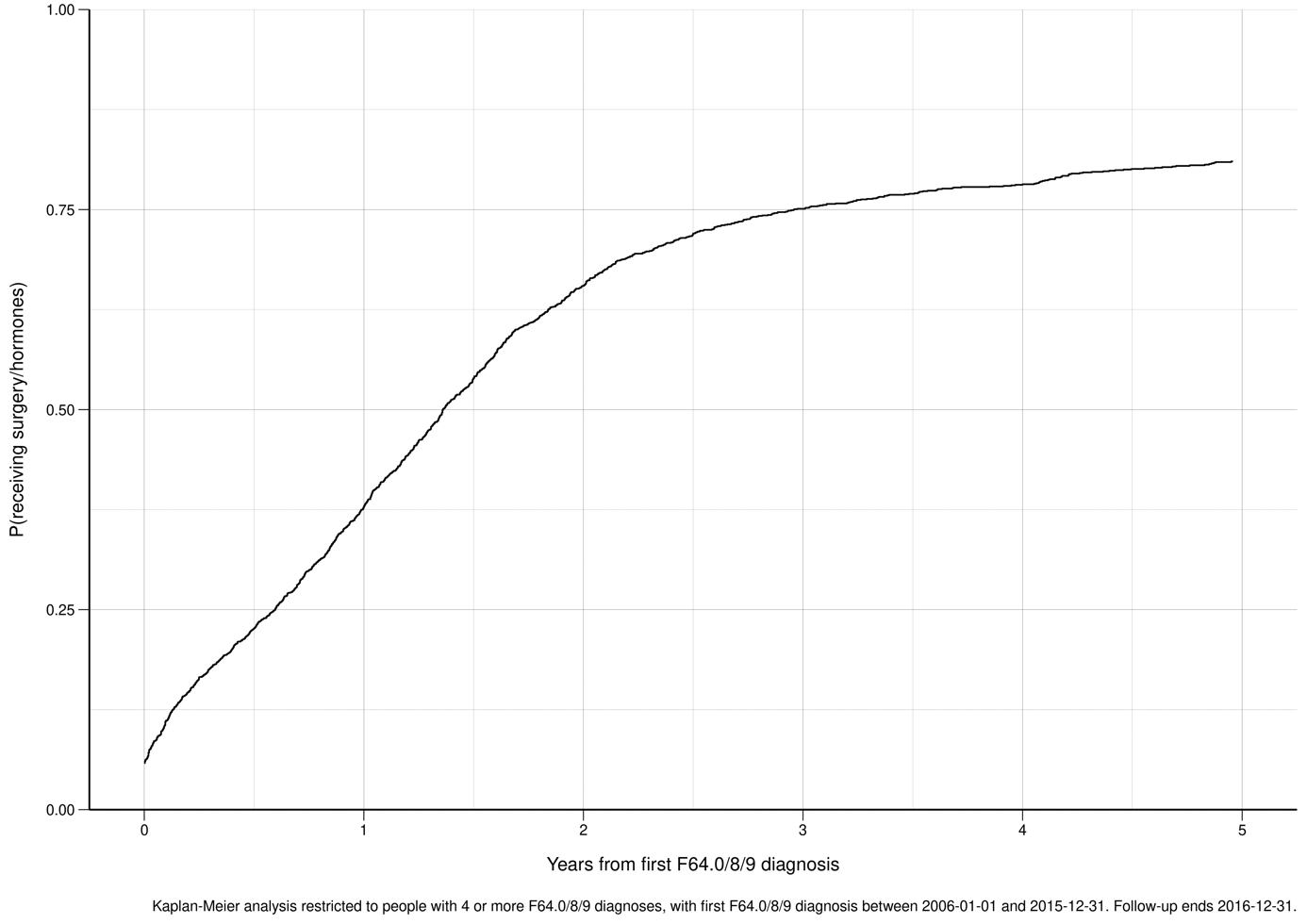


| **Supplementary table 1: Coverage in outpatient registers** |  |  |
| --- | --- | --- |

| **Year** | **Number of visits, psychiatry, MD**  (data from hospitals, SKR) | **Number of registered visits, psychiatry, MD** (data from NPR) | **Number of visits with missing diagnoses in the NPR** | **Proportion of registered diagnoses** |
| --- | --- | --- | --- | --- |
| **2001** | 916600 | 379704 | 306385 | 8,0 |
| **2002** | 950900 | 556232 | 422362 | 14,1 |
| **2003** | 925300 | 530089 | 366005 | 17,7 |
| **2004** | 937700 | 599158 | 343966 | 27,2 |
| **2005** | 937900 | 636596 | 353580 | 30,2 |
| **2006** | 933 390 | 681867 | 245631 | 46,7 |
| **2007** | 946 447 | 637852 | 128045 | 53,9 |
| **2008** | 978 061 | 703268 | 120197 | 59,6 |
| **2009** | 1 015 605 | 767471 | 141516 | 61,6 |
| **2010** | 1 046 236 | 770268 | 126472 | 61,5 |
| **2011** | 1 087 956 | 887627 | 134803 | 69,2 |
| **2012** | 1 095 486 | 942705 | 120696 | 75,0 |
| **2013** | 1 097 467 | 972476 | 75724 | 81,7 |
| **2014** | 1 119 414 | 1008216 | 63960 | 84,4 |
| **2015** | 1 134 915 | 1167583 | 115988 | 92,7 |
| **2016** | 1 148 552 | 1101946 | 73522 | 89,5 |
| **2017** | 1 164 979 | 1144132 | 66900 | 92,5 |
| **2018** | 1 147 474 | 1148140 | 55973 | 95,2 |
| **2019** | 1 137 246 | 1177492 | 57779 | 98,5 |

| Assigned sex  at birth | Diagnoses  (n) | Individuals  (n) | Individuals with  GCMI (n) | Individuals with  GCMI (%) | Diagnoses  (n) | Individuals  (n, cumulative) | Individuals with GCMI  (n, cumulative) | Individuals with GCMI (%,cumulative) |
| --- | --- | --- | --- | --- | --- | --- | --- | --- |
| Assigned female | 1 diagnosis | 70 | 3 | 4,3 | ≥ 1 diagnosis | 1108 | 816 | 73,6 |
| Assigned female | 2 diagnoses | 46 | 6 | 13 | ≥ 2 diagnoses | 1038 | 813 | 78,3 |
| Assigned female | 3 diagnoses | 33 | 9 | 27,3 | ≥ 3 diagnoses | 992 | 807 | 81,4 |
| Assigned female | 4 diagnoses | 36 | 9 | 25 | ≥ 4 diagnoses | 959 | 798 | 83,2 |
| Assigned female | 5 diagnoses | 31 | 13 | 41,9 | ≥ 5 diagnoses | 923 | 789 | 85,5 |
| Assigned female | 6 diagnoses | 50 | 26 | 52 | ≥ 6 diagnoses | 892 | 776 | 87 |
| Assigned female | 7 diagnoses | 51 | 36 | 70,6 | ≥ 7 diagnoses | 842 | 750 | 89,1 |
| Assigned female | 8 diagnoses | 60 | 48 | 80 | ≥ 8 diagnoses | 791 | 714 | 90,3 |
| Assigned female | 9 diagnoses | 55 | 45 | 81,8 | ≥ 9 diagnoses | 731 | 666 | 91,1 |
| Assigned female | ≥ 10 diagnoses | 676 | 621 | 91,9 | ≥ 10 diagnoses | 676 | 621 | 91,9 |
| Assigned male | 1 diagnosis | 113 | 5 | 4,4 | ≥ 1 diagnosis | 978 | 592 | 60,5 |
| Assigned male | 2 diagnoses | 47 | 6 | 12,8 | ≥ 2 diagnoses | 865 | 587 | 67,9 |
| Assigned male | 3 diagnoses | 38 | 5 | 13,2 | ≥ 3 diagnoses | 818 | 581 | 71 |
| Assigned male | 4 diagnoses | 38 | 11 | 28,9 | ≥ 4 diagnoses | 780 | 576 | 73,8 |
| Assigned male | 5 diagnoses | 35 | 17 | 48,6 | ≥ 5 diagnoses | 742 | 565 | 76,1 |
| Assigned male | 6 diagnoses | 43 | 24 | 55,8 | ≥ 6 diagnoses | 707 | 548 | 77,5 |
| Assigned male | 7 diagnoses | 43 | 27 | 62,8 | ≥ 7 diagnoses | 664 | 524 | 78,9 |
| Assigned male | 8 diagnoses | 38 | 29 | 76,3 | ≥ 8 diagnoses | 621 | 497 | 80 |
| Assigned male | 9 diagnoses | 44 | 28 | 63,6 | ≥ 9 diagnoses | 583 | 468 | 80,3 |
| Assigned male | ≥ 10 diagnoses | 539 | 440 | 81,6 | ≥ 10 diagnoses | 539 | 440 | 81,6 |

**Supplementary Table 2a.** Numbers and percentages of individuals with GCMI by occurrences of GD diagnoses, 2006-2014.

| Assigned sex  at birth | Diagnoses  (n) | Individuals  (n) | Individuals with  GCMI (n) | Individuals with  GCMI (%) | Diagnoses  (n) | Individuals  (n, cumulative) | Individuals with GCMI  (n, cumulative) | Individuals with GCMI (%, cumulative) |
| --- | --- | --- | --- | --- | --- | --- | --- | --- |
| Assigned female | 1 diagnosis | 30 | 2 | 6,7 | ≥ 1 diagnosis | 307 | 162 | 52,8 |
| Assigned female | 2 diagnoses | 12 | 0 | 0 | ≥ 2 diagnoses | 277 | 161 | 58,1 |
| Assigned female | 3 diagnoses | 8 | 3 | 37,5 | ≥ 3 diagnoses | 265 | 161 | 60,8 |
| Assigned female | 4 diagnoses | 9 | 2 | 22,2 | ≥ 4 diagnoses | 257 | 159 | 61,9 |
| Assigned female | 5 diagnoses | 16 | 5 | 31,2 | ≥ 5 diagnoses | 248 | 157 | 63,3 |
| Assigned female | 6 diagnoses | 17 | 5 | 29,4 | ≥ 6 diagnoses | 232 | 154 | 66,4 |
| Assigned female | 7 diagnoses | 21 | 11 | 52,4 | ≥ 7 diagnoses | 215 | 149 | 69,3 |
| Assigned female | 8 diagnoses | 18 | 14 | 77,8 | ≥ 8 diagnoses | 194 | 140 | 72,2 |
| Assigned female | 9 diagnoses | 10 | 8 | 80 | ≥ 9 diagnoses | 176 | 131 | 74,4 |
| Assigned female | ≥ 10 diagnoses | 166 | 149 | 89,8 | ≥ 10 diagnoses | 166 | 125 | 75,3 |
| Assigned male | 1 diagnosis | 45 | 2 | 4,4 | ≥ 1 diagnosis | 294 | 144 | 49 |
| Assigned male | 2 diagnoses | 17 | 3 | 17,6 | ≥ 2 diagnoses | 249 | 142 | 57 |
| Assigned male | 3 diagnoses | 16 | 2 | 12,5 | ≥ 3 diagnoses | 232 | 139 | 59,9 |
| Assigned male | 4 diagnoses | 17 | 4 | 23,5 | ≥ 4 diagnoses | 216 | 137 | 63,4 |
| Assigned male | 5 diagnoses | 11 | 5 | 45,5 | ≥ 5 diagnoses | 199 | 133 | 66,8 |
| Assigned male | 6 diagnoses | 24 | 13 | 54,2 | ≥ 6 diagnoses | 188 | 128 | 68,1 |
| Assigned male | 7 diagnoses | 19 | 13 | 68,4 | ≥ 7 diagnoses | 164 | 115 | 70,1 |
| Assigned male | 8 diagnoses | 12 | 5 | 41,7 | ≥ 8 diagnoses | 145 | 102 | 70,3 |
| Assigned male | 9 diagnoses | 8 | 5 | 62,5 | ≥ 9 diagnoses | 133 | 97 | 72,9 |
| Assigned male | ≥ 10 diagnoses | 125 | 92 | 73,6 | ≥ 10 diagnoses | 125 | 92 | 73,6 |

**Supplementary Table 2b.** Numbers and percentages of individuals with GCMI by occurrences of GD diagnoses, 2006-2009

**Supplementary Table 2c.** Numbers and percentages of individuals with GCMI by occurrences of GD diagnoses, 2010-2014

| Assigned sex  at birth | Diagnoses  (n) | Individuals  (n) | Individuals with  GCMI (n) | Individuals with  GCMI (%) | Diagnoses  (n) | Individuals  (n, cumulative) | Individuals with GCMI  (n, cumulative) | Individuals with GCMI (%, cumulative) |
| --- | --- | --- | --- | --- | --- | --- | --- | --- |
| Assigned female | 1 diagnosis | 47 | 1 | 2,1 | ≥ 1 diagnosis | 801 | 592 | 73,9 |
| Assigned female | 2 diagnoses | 37 | 5 | 13,5 | ≥ 2 diagnoses | 754 | 591 | 78,4 |
| Assigned female | 3 diagnoses | 31 | 9 | 29 | ≥ 3 diagnoses | 717 | 586 | 81,7 |
| Assigned female | 4 diagnoses | 32 | 8 | 25 | ≥ 4 diagnoses | 686 | 577 | 84,1 |
| Assigned female | 5 diagnoses | 28 | 11 | 39,3 | ≥ 5 diagnoses | 654 | 569 | 87 |
| Assigned female | 6 diagnoses | 38 | 23 | 60,5 | ≥ 6 diagnoses | 626 | 558 | 89,1 |
| Assigned female | 7 diagnoses | 40 | 31 | 77,5 | ≥ 7 diagnoses | 588 | 535 | 91 |
| Assigned female | 8 diagnoses | 47 | 41 | 87,2 | ≥ 8 diagnoses | 548 | 504 | 92 |
| Assigned female | 9 diagnoses | 50 | 44 | 88 | ≥ 9 diagnoses | 501 | 463 | 92,4 |
| Assigned female | ≥ 10 diagnoses | 451 | 419 | 92,9 | ≥ 10 diagnoses | 451 | 419 | 92,9 |
| Assigned male | 1 diagnosis | 75 | 3 | 4 | ≥ 1 diagnosis | 684 | 425 | 62,1 |
| Assigned male | 2 diagnoses | 42 | 6 | 14,3 | ≥ 2 diagnoses | 609 | 422 | 69,3 |
| Assigned male | 3 diagnoses | 29 | 5 | 17,2 | ≥ 3 diagnoses | 567 | 416 | 73,4 |
| Assigned male | 4 diagnoses | 31 | 9 | 29 | ≥ 4 diagnoses | 538 | 411 | 76,4 |
| Assigned male | 5 diagnoses | 30 | 16 | 53,3 | ≥ 5 diagnoses | 507 | 402 | 79,3 |
| Assigned male | 6 diagnoses | 37 | 20 | 54,1 | ≥ 6 diagnoses | 477 | 386 | 80,9 |
| Assigned male | 7 diagnoses | 28 | 17 | 60,7 | ≥ 7 diagnoses | 440 | 366 | 83,2 |
| Assigned male | 8 diagnoses | 30 | 25 | 83,3 | ≥ 8 diagnoses | 412 | 349 | 84,7 |
| Assigned male | 9 diagnoses | 31 | 23 | 74,2 | ≥ 9 diagnoses | 382 | 324 | 84,8 |
| Assigned male | ≥ 10 diagnoses | 351 | 301 | 85,8 | ≥ 10 diagnoses | 351 | 301 | 85,8 |

**Supplementary table 3:** Percentual differences in incidence rates by ≥1 diagnosis, ≥4 diagnoses, ≥1 diagnosis with GCMI in 2001-2015, 2006-2010 and 2011-2015.

| Assigned sex | Age category | Time period | Diagnoses  (≥1) | Diagnoses  (≥4) | Diagnoses (≥1)  + treatment | % difference between diagnosis (≥1)/diagnosis (≥4) | % difference between diagnosis (≥1)/  diagnosis (≥1)  treatment |
| --- | --- | --- | --- | --- | --- | --- | --- |
| Assigned female | [10,18] | 2001-2005* | 8 | 7 | 5 | 14% | 60% |
| Assigned female | [10,18] | 2006-2010 | 66 | 63 | 60 | 5% | 10% |
| Assigned female | [10,18] | 2011-2015 | 276 | 205 | 176 | 35% | 57% |
| Assigned female | (18,30] | 2001-2005 | 102 | 84 | 76 | 21% | 34% |
| Assigned female | (18,30] | 2006-2010 | 259 | 226 | 198 | 15% | 31% |
| Assigned female | (18,30] | 2011-2015 | 611 | 483 | 421 | 27% | 45% |
| Assigned female | (30,50] | 2001-2005 | 71 | 56 | 44 | 27% | 61% |
| Assigned female | (30,50] | 2006-2010 | 106 | 92 | 74 | 15% | 43% |
| Assigned female | (30,50] | 2011-2015 | 142 | 119 | 94 | 19% | 51% |
| Assigned female | (51-] | 2001-2005 | 28 | 14 | 15 | 100% | 87% |
| Assigned female | (51- ] | 2006-2010 | 32 | 24 | 14 | 33% | 129% |
| Assigned female | (51-] | 2011-2015 | 31 | 19 | 18 | 63% | 72% |
| Assigned male | [10,18] | 2001-2005 | 22 | 16 | 16 | 38% | 38% |
| Assigned male | [10,18] | 2006-2010 | 24 | 21 | 21 | 14% | 14% |
| Assigned male | [10,18] | 2011-2015 | 88 | 64 | 51 | 38% | 73% |
| Assigned male | (18,30] | 2001-2005 | 125 | 92 | 89 | 36% | 40% |
| Assigned male | (18,30] | 2006-2010 | 211 | 180 | 125 | 17% | 69% |
| Assigned male | (18,30] | 2011-2015 | 571 | 435 | 364 | 31% | 57% |
| Assigned male | (30,50] | 2001-2005 | 133 | 105 | 105 | 27% | 27% |
| Assigned male | (30,50] | 2006-2010 | 149 | 119 | 88 | 25% | 69% |
| Assigned male | (30,50] | 2011-2015 | 182 | 126 | 100 | 44% | 82% |
| Assigned male | (51-] | 2001-2005 | 68 | 37 | 46 | 84% | 48% |
| Assigned male | (51-] | 2006-2010 | 73 | 48 | 53 | 52% | 38% |
| Assigned male | (51-] | 2011-2015 | 82 | 54 | 55 | 52% | 49% |
| * The differences in rates between >1 diagnosis and >1 diagnosis + treatment in the period 2001-2005 are not directly comparable to those in 2006-2010 and 2011-2015, due to the fact that the prescription drugs register started on mid 2005. People on hormonal treatment only during 2001-2005 may be misclassified as not receiving gender affirming medical treatment. | | | | | | | |

**Supplementary Figure 2.** Crude (A) and coverage-adjusted (B) incidence rates of GD, defined as ≥ 4 diagnoses, in Sweden 2001-2015, stratified by birth assigned sex and age.

**A.**


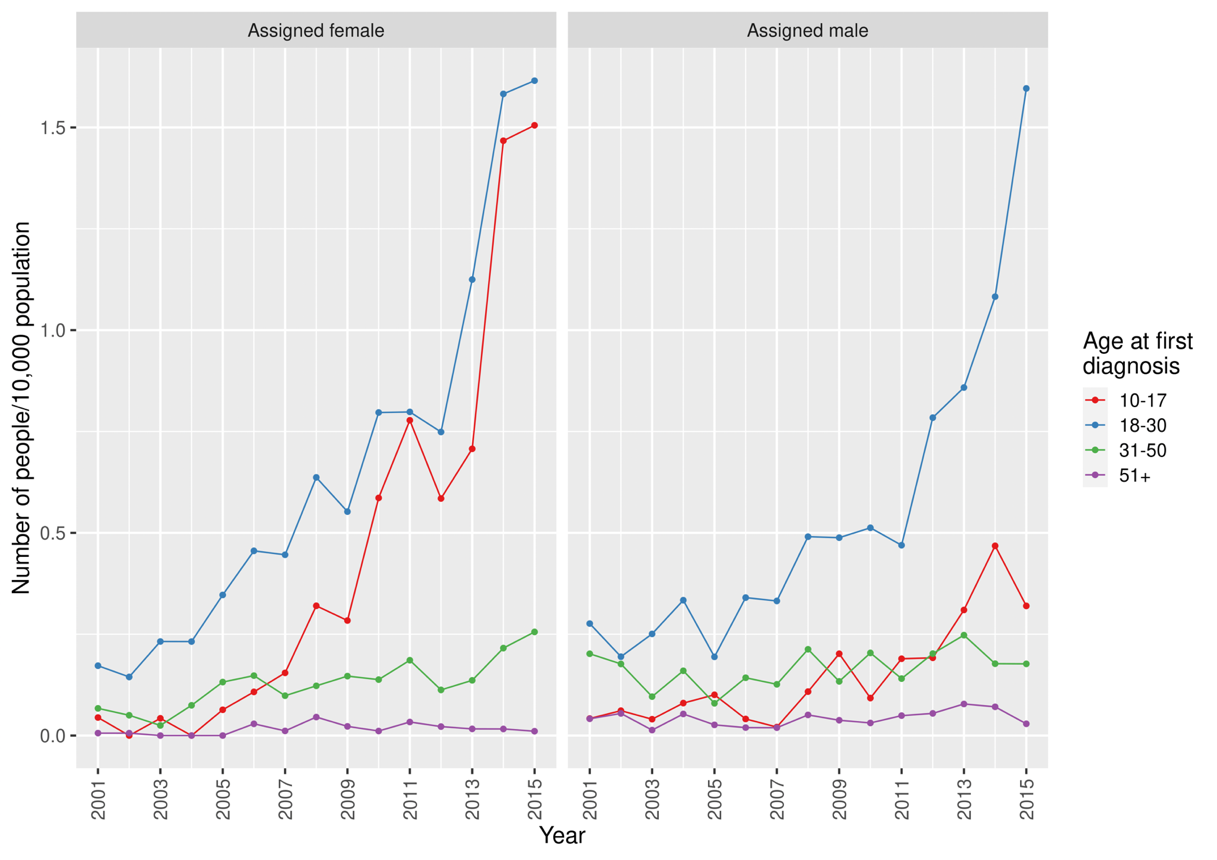


**B.**


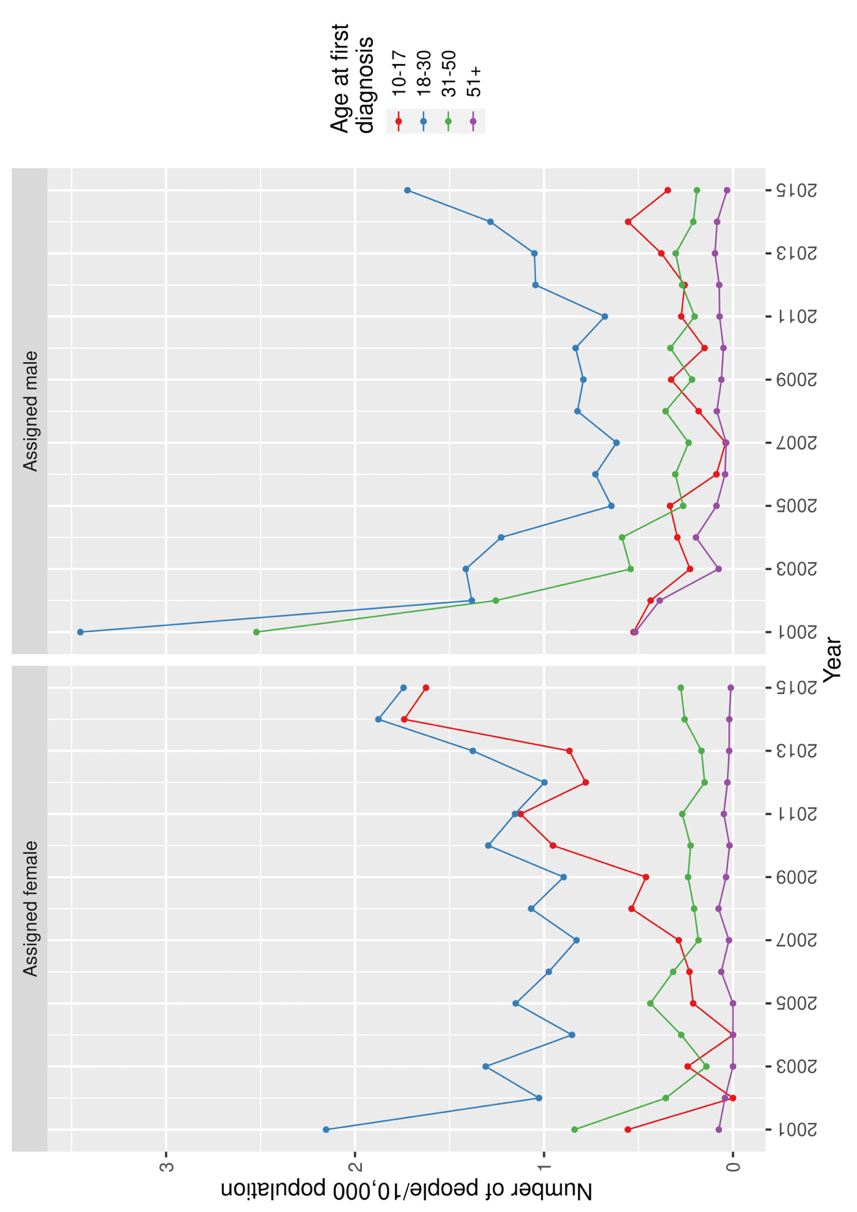


**Supplementary Table 5.** Crude and coverage-adjusted Poisson-derived incidence rate ratios (IRR) with 95% confidence intervals by sex and age

groups as well as interaction terms for time trends and assigned sex, in Sweden 2001-2015.

|  | Crude IRR | 95% CI | P-value for interaction term time*assigned sex | Adjusted  IRR | 95% CI | P-value for interaction term time*assigned sex |
| --- | --- | --- | --- | --- | --- | --- |
| Age 10-17 |  |  | <0,001 |  |  | <0,001 |
| aF | 1.34 | 1.29-1.39 |  | 1.22 | 1.17-1.27 |  |
| aM | 1.19 | 1.13-1.26 |  | 1.07 | 1.01-1.13 |  |
| Age 18-30 |  |  | 0.29 |  |  | 0.26 |
| aF | 1.18 | 1.15-1.20 |  | 1.05 | 1.03-1.08 |  |
| aM | 1.16 | 1.14-1.18 |  | 1.03 | 1.01-1.06 |  |
| Age 31-50 |  |  | <0,001 |  |  | <0.001 |
| aF | 1.10 | 1.06-1.13 |  | 0.97 | 0.93-1.00 |  |
| aM | 1.02 | 1.00-1.05 |  | 0.89 | 0.86-0.91 |  |
| Age 51+ |  |  | 0.38 |  |  | 0.34 |
| aF | 1.08 | 1.00-1.16 |  | 0.95 | 0.88-1.03 |  |
| aM | 1.04 | 0.99-1.09 |  | 0.91 | 0.86-0.96 |  |
